# Phage therapy for *Klebsiella pneumoniae*: Understanding bacteria–phage interactions for therapeutic innovations

**DOI:** 10.1371/journal.ppat.1012971

**Published:** 2025-04-08

**Authors:** Julie Le Bris, Nathalie Chen, Adeline Supandy, Olaya Rendueles, Daria Van Tyne

**Affiliations:** 1 Institut Pasteur, Université Paris Cité, CNRS UMR3525, Microbial Evolutionary Genomics, Paris, France; 2 Sorbonne Université, Collège Doctoral, Ecole Doctorale Complexité du Vivant, Paris, France; 3 Division of Infectious Diseases, University of Pittsburgh School of Medicine, Pittsburgh, Pennsylvania, United States of America; 4 Laboratoire de Microbiologie et Génétique Moléculaires (LMGM), CNRS UMR5100, Centre de Biologie Intégrative (CBI), Université de Toulouse, CNRS, Université Toulouse III - Paul Sabatier (UT3), Toulouse, France; 5 Center for Evolutionary Biology and Medicine, University of Pittsburgh School of Medicine, Pittsburgh, Pennsylvania, United States of America; University of Virginia, UNITED STATES OF AMERICA

## Abstract

*Klebsiella pneumoniae* (KP) is a Gram-negative bacterium that commonly resides in the human gastrointestinal tract and can also act as an opportunistic pathogen and cause extra-intestinal infections. KP poses a global health threat because it causes both hospital- and community-acquired infections in immune-competent and immunocompromised hosts. These infections can be multidrug-resistant and/or hypervirulent, making KP infections difficult to treat and deadly. In the absence of effective treatments for recalcitrant KP infections, bacteriophage (phage) therapy is gaining attention as a promising alternative. In this review, we evaluate KP epidemiology and epitope diversity, discuss interactions between KP-targeting phages and their bacterial hosts from an eco-evolutionary perspective, and summarize recent efforts in phage therapy for treating KP infections. We also discuss novel approaches, including genetic engineering and machine learning, as initial steps toward developing KP-targeting phage therapy as a precision medicine approach for an emerging and dangerous pathogen.

## Introduction

**Fig 1:**
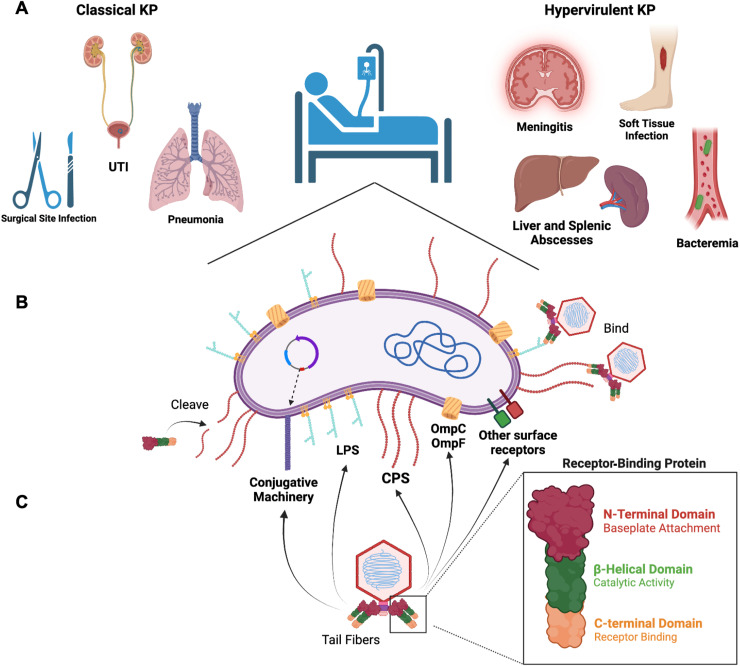
*Klebsiella pneumoniae* infections and cell surface phage receptors. **(A)** Classical KP strains are typically associated with pneumonia, urinary tract infections (UTIs), and surgical site infections. Hypervirulent KP strains are associated with invasive infections such as meningitis, soft tissue infections, liver and splenic abscesses, and bacteremia. **(B)** The primary cell surface receptor for KP phage is the capsular polysaccharide (CPS). Other phage receptors include lipopolysaccharide (LPS), outer membrane porins (OMPs), and conjugative plasmid-encoded pili. **(C)** Phages bind to cell surface receptors using receptor-binding proteins on their tail fibers. These proteins can contain catalytic domains (e.g., depolymerase domains) that aid in targeting cell surface receptors. This figure was created using BioRender.

*Klebsiella pneumoniae* (KP) are gut commensals that can also cause opportunistic infections. KP can be categorized into two distinct pathotypes, called classical and hypervirulent. Classical strains are associated with infections in hospitalized and immunocompromised patients, are frequently multidrug-resistant, and cause hospital-associated infections such as urinary tract infections, pneumonia, and surgical site infections (Fig 1A) [1]. In contrast, hypervirulent KP strains are usually community-acquired, infect healthy individuals, are often susceptible to antibiotics, and are able to cause highly invasive infections like liver and splenic abscesses, endophthalmitis, and meningitis (Fig 1A) [1]. While genomic studies have shown that the classical and hypervirulent KP pathotypes have followed independent evolutionary trajectories [2], recent studies report a worrisome convergence of multidrug-resistant and hypervirulent traits in some strains [3]. This poses a challenge, as the pace of new antimicrobial discovery and approval has not kept up with the increasing emergence and spread of high-risk KP clones [4].

To fight high-risk KP clones, therapeutic strategies have been developed to target different surface antigens, including capsular polysaccharide (CPS) and lipopolysaccharide (LPS) [5,6]. While vaccination and antibody therapies have been a primary focus in recent decades [7,8], bacteriophage (hereafter referred to as phage) therapy is gaining widespread attention as a new approach for treating KP infections [9]. Phages are bacteria-targeting viruses that can be used to treat infections. Naturally occurring phages and their bacterial hosts are constantly entangled in an evolutionary arms race, and prior work has suggested that the diversity of bacterial antigens like CPS and LPS is likely driven by phage predation [10,11]. Understanding the eco-evolutionary dynamics between phages and bacterial antigens is critical in the deployment of phage therapy as a successful alternative therapeutic strategy for treating infections, like those caused by KP. Here, we review the diversity of surface antigens recognized by KP-targeting phages, analyze phage-host dynamics, explore the use of phage therapy to treat KP infections, and discuss how phages could be further harnessed as an alternative antimicrobial strategy.

## *Klebsiella pneumoniae* surface polysaccharides

KP is well known for producing a CPS, which is a major contributor to its virulence [12]. CPS is attached to the outer membrane with a lipid anchor, but may also be retained at the bacterial surface through interactions with other surface molecules such as LPS [13]. The KP CPS is composed of repeating oligosaccharide units that together form the K-antigen, which defines the K-type of a KP strain. In this review, the terms K-antigen and CPS will be used interchangeably. CPS can be composed of a variety of different carbohydrates including glucose, galactose, galactofuranose, fucose, mannose, and rhamnose [14]. These sugar moieties may be additionally modified by CPS-modifying enzymes such as acetyltransferases and pyruvyl transferases [14]. CPS composition is diverse and varies between different KP strains. Additionally, hypervirulent strains typically produce more CPS than classical strains and are often found to be hypermucoviscous [1]. The other dominant surface-associated polysaccharide, LPS, is composed of a lipid A molecule embedded in the bacterial outer membrane, a core oligosaccharide domain, and a variable O-antigen made of repeating sugar units that is used to define the O-antigen type. The O-antigen is composed of sugars such as galactose, galactofuranose, mannose, ribofuranose, and N-acetyl-d-glucosamine [15] and can be additionally modified (e.g., acetylated) to generate subvariants of O-antigens [16,17].

Typing and tracking of K-antigens and O-antigens can help identify which antigen types are more commonly associated with KP infections, and thus identify the K-types and O-types that should be prioritized for the development of new therapeutics. Historically, both CPS and LPS were typed using antisera reactive to specific and immunologically defined K-antigen or O-antigen types termed serotypes. K-antigen typing was initially performed with the Quellung capsular swelling reaction, in which typing serum is added to bacteria and then observed under a microscope for capsular swelling, which happens upon binding of type-specific antibodies to the K-antigen [18]. Several other methods were later developed to increase speed, accuracy, and efficiency, including indirect immunofluorescence, slide agglutination, double-diffusion gel precipitation (Ouchterlony test), countercurrent immunoelectrophoresis, and latex agglutination [19–21]. However, the requirement for antisera to perform these tests limited comprehensive identification, and many KP isolates were unable to be typed due to the limited number of antisera available. Additionally, cross-reactivity between different serotypes made precise identification challenging. Like K-antigen typing, O-antigen typing was also traditionally performed using antisera in a tube or latex agglutination test [22], but this had the additional challenge of requiring acapsular mutants, as the K-antigen often masks the O-antigen. An enzyme-linked immunosorbent assay that did not require acapsular mutants was later developed, thereby facilitating the O-antigen typing process [23].

More recently, genetic methods based on PCR or whole genome sequencing (WGS) were developed for both K-antigen and O-antigen typing. PCR-based methods include restriction fragment length polymorphism analysis of the entire CPS locus to determine a “C-pattern,” and typing based on the sequences of specific CPS biosynthesis genes such as *wzi*, *wzc*, and *wzy* [24–26]. Similarly, O-antigen typing can be performed using PCR to identify specific alleles in the *wzm*-*wzt* genes in the O-antigen locus, as well as alleles in the *wbbY* region [27]. As WGS has become more accessible in recent years, genomic typing of K-antigen and O-antigen loci is now preferred as it is more precise and comprehensive. Software tools like Kaptive have been instrumental in the development of a standardized typing scheme for KP isolates, even in the presence of genetic mutations and locus disruptions [28,29]. Kaptive is regularly updated and currently enables the identification of 163 genetically defined K-antigen types (also called K-loci) and 11 different O loci [28].

Despite the ability to assign K-antigen types from WGS data, additional work is still needed to link K-antigen locus genotypes to biochemical structures, as these cannot be predicted based solely on genomic sequence. Among the 163 different K-antigen types, only about half have a determined structure [14,30,31]. While K-antigen structures have historically been identified using gas chromatography-mass spectrometry and nuclear magnetic resonance spectroscopy, a recent study used Fourier transform infrared spectroscopy to predict K-antigen structure based on similarity to known K-antigens [32]. As more structures are elucidated, it is tempting to speculate that one day new K-antigen types could be inferred from genome sequencing data alone, though biochemical validation would still be necessary to confirm polysaccharide composition.

## CPS epidemiology and impact on virulence

**Fig 2:**
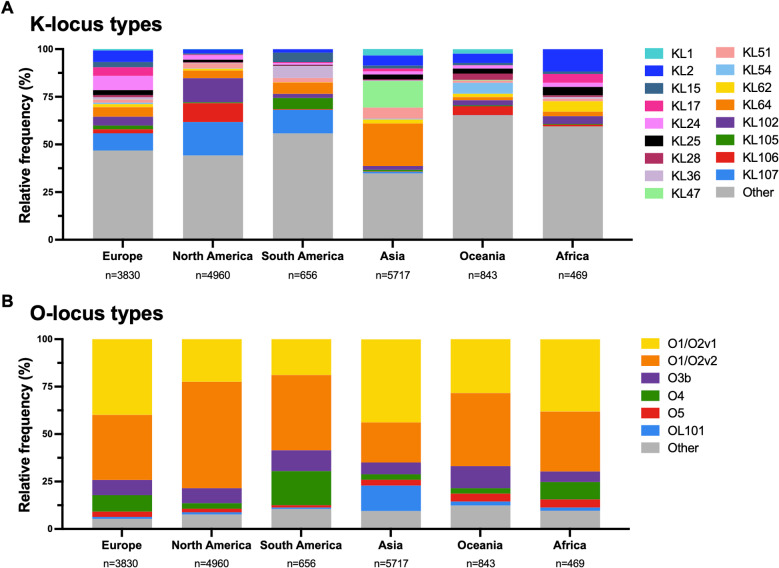
K- and O- locus type diversity across different geographic regions. 16,475 KP genomes derived from human blood, urinary tract, or respiratory specimens were accessed from NCBI on October 16, 2023. Genomes were typed using Kaptive to determine K-locus types **(A)** and O-locus types **(B)** [28,29]. The most prevalent types are labeled; all other types are grouped as “Other.” K-loci and O-loci flagged as “unknown” by Kaptive are included in the “Other” category.

The high diversity of KP CPS types appears to be a major determinant of the adaptive success of KP, and the distribution of K-antigen types varies by geography [33,34]. Common K-antigen types described in the literature include KL2, KL10, KL15, KL16, KL17, KL21, KL22, KL24, KL25, KL28, KL30, KL54, KL62, and KL64. For this review, we surveyed 16,475 KP genomes deposited in NCBI (accessed October 16, 2023) that were collected from humans and sampled from urine, blood, or the respiratory tract. Among these genomes, we found that the ten most frequently observed K-loci were KL2, KL24, KL25, KL47, KL51, KL64, KL102, KL106, KL107, and KL112. We also observed enrichment of different K-loci on different continents, confirming regional differences in prevalence (Fig 2A).

CPS is an important virulence factor for KP [12]. Experimental disruption of CPS in a variety of KP strains has been shown to decrease virulence in mouse models of infection compared to encapsulated parent strains [35,36]. CPS has also been shown to mediate evasion of phagocytosis and complement-mediated lysis [37,38], and limits the inflammatory response to KP infection [36]. Beyond simply the presence of CPS, the amount and composition of the CPS also impact KP virulence. For example, CPS associated with hypervirulent strains is often hypermucoviscous, and this characteristic has been demonstrated to correlate with increased virulence [12,39]. Hypermucoviscosity is associated with specific K-antigen types, including KL1, KL2, KL4, and KL5 [40]. Of these, KL1 and KL2 have been particularly well characterized and shown to confer hypervirulence, defined as causing lethal infection in mice at a low bacterial inoculum (10^3^ bacteria) and the ability to cause disease in otherwise healthy humans. KL1 and KL2 antigen types have also been linked to more invasive disease and increased resistance to phagocytosis, killing by neutrophils, and capture by liver-resident macrophages [41–44]. The ability of CPS loci to be horizontally transferred between genetically distinct KP strains has caused some researchers to propose that KP virulence is associated with particular genetic lineages rather than specific K-antigen types [40,45]. To rigorously study CPS-specific mechanisms without confounding from the genetic background, some recent studies have performed CPS swap experiments. Notably, Huang and colleagues found that “high-virulence” K-antigen types conferred the ability to evade capture by liver-resident macrophages more than “low-virulence” types [42]. However, other studies have shown that the transfer of a hypervirulent CPS to a less virulent strain does not fully recapitulate virulence [46–48]. Additionally, it is not uncommon to observe clinical KP strains with disruptions in CPS biosynthesis genes such as *wcaJ* and *wbaP* [49,50], further highlighting the complex relationship between K-antigen type and KP virulence.

## Lipopolysaccharide structure, epidemiology, and associated virulence

In contrast to the 163 K-antigen types currently known, only 11 primary O-antigen types have been described, including O1, O2a, O2ac, O2aeh, O3, O4, O5, O7, O8, O11, and O12 [16]. All except O11 have published structures [31], and four additional types (OL101, OL102, OL103, and OL104) have been genetically identified but not yet structurally characterized [33]. One final O-antigen type, O2afg, is associated with the ST258 lineage and is also considered to be a distinct type [16,51]. Unlike K-antigens, whose structure is dictated entirely by the K-locus, O-antigen structures are determined by both the O-locus and additional genes outside the locus (*wbbY*, *gmlABD*, and *wbVW*) [28]. For example, the WbbY glycosyltransferase modifies the O1 antigen type and converts it to the O2 antigen type. Additionally, several O-antigens are structurally similar and differ by the presence of an additional subunit or by a modification such as acetylation, resulting in O-antigen subvariants [16,17,52]. Of the different O-antigen types, only a few are commonly found in clinical KP strains. Four O-antigen types, O1, O2ab, O3, and O5, accounted for 82% of strains tested in two German university hospitals and 92% of human-derived strains tested in Japan [22,53]. In line with these seroepidemiological studies, among the 16,475 KP genomes we accessed from NCBI, we found that the O1/O2v1 and O1/O2v2 loci were the most common O-loci, followed by O3b, OL101, O4, and O5. There was less regional variation in O-loci compared to K-loci, however, we did find the O4 locus to be more common in South America and the OL101 locus to be predominantly found in Asia (Fig 2B).

LPS, specifically lipid A, is strongly immunogenic and an activator of the pattern recognition receptor TLR4 [12,54]. Some KP strains are able to dampen this immunogenicity by masking LPS with specific CPS antigens [12,38]. LPS has also been implicated in the virulence of KP and contributes to bacterial resistance to complement-mediated killing by binding complement protein C3b far from the cell membrane and thus preventing the formation and insertion of the membrane attack complex [55]. The O1 serotype in particular is associated with more invasive and hypervirulent strains [28], and contributes to bacteremia in a murine model of pneumonia [56]. Finally, lipid A may also contribute to virulence by conferring protection against cationic antimicrobial peptides [12,57]. Overall, while LPS is more immunogenic than CPS, both CPS and LPS are highly abundant surface polysaccharides that contribute to KP virulence in different ways.

## Diversity of KP surface receptors from a phage therapy perspective

Antibiotics are currently the first line of treatment against KP infections. Due to the quick acquisition of antibiotic resistance by KP [58], antibiotics are no longer effective in clearing some infections and alternative approaches are required. Phage therapy is gaining attention as an alternative treatment for antibiotic-resistant bacterial infections. In contrast to broad-spectrum antimicrobials, phage therapy can specifically target pathogens and preserve beneficial bacteria in the microbiome [59,60], sparing patients from the microbial dysbiosis that can accompany antibiotic treatment.

KP-targeting phages have been isolated from a broad range of sources where KP bacteria are prevalent, including water, soil, and clinical samples [61]. The first KP phage was identified over a hundred years ago [62], and since then, more than 10,000 have been isolated [63]. KP phages belong mainly to the *Caudoviricetes* class of viruses [64], which are tailed viruses with double-stranded DNA (dsDNA) genomes ranging in size from 5 to 300+ kilobases [65]. *Caudoviricetes* phages are composed of (i) a head or capsid, which encases the dsDNA, (ii) a helical tail that injects DNA into the bacterial cytoplasm, (iii) a portal complex which links the head and tail, and (iv) tail fiber and tailspike proteins attached to the baseplate which interact with bacterial cell surface receptors to initiate infection. Until recently, phages were classified by their morphological characteristics, with tailed phages belonging to three families: myoviruses (long contractile tails), siphoviruses (long non-contractile tails), and podoviruses (short non-contractile tails) [66]. However, this classification scheme did not accurately reflect the evolutionary history of phages, and a new genome-based classification was recently proposed by the International Committee on the Taxonomy of Viruses [64]. Despite the significant number of phages that remain to be classified, *Caudoviricetes* is now divided into 47 different families. According to this new classification, phages infecting KP are distributed across the phylogeny of dsDNA phages, with most belonging to the *Ackermannviridae*, *Autographiviridae, Demerecviridae*, *Drexlerviridae*, and *Straboviridae* families [67,68]. Recently, an open-source expandable phage and strain collection, known as KlebPhaCol, was initiated to collect, store, and distribute *Klebsiella* spp. bacterial strains and phages [69]. As researchers continue to study *Klebsiella*-targeting phages, their known diversity is likely to increase accordingly.

The initial steps in phage infection of bacterial cells involve the recognition of surface receptors and subsequent phage adsorption (i.e., binding) to the cell. These first steps are required for productive infection and are the primary determinants of the range of hosts that a particular phage can infect. Initiation of phage infection can happen in a single step by irreversible binding to a receptor [66], or in two steps, where initial reversible binding to a primary receptor is followed by irreversible binding to a secondary surface protein in proximity to the bacterial membrane [70]. Because the presence of a host cell receptor and irreversible binding are required for the release of phage genetic material into the cell, phages use highly variable receptor-binding proteins (RBPs) that recognize specific bacterial surface receptors to initiate infection (Fig 1C). The presence of suitable surface receptors, however, does not guarantee a successful infection because many bacteria encode genome defense systems that have evolved to protect against phage predation. In recent years, a myriad of additional anti-phage defense systems have been described [71,72]. On average, a KP genome encodes six anti-phage defense systems and they are often non-redundant [73]. These defense systems include restriction-modification [74], CRISPR-Cas [75], and abortive infection [76], among others. In the context of phage therapy, and for this review, only productive infection, whereby phages replicate and produce infectious viral progeny, will be considered. Other viral infection strategies like lysogeny or pseudolysogeny are not considered here but are reviewed elsewhere [77].

## Bacterial epitopes as receptors for *K. pneumoniae* phages

Because of its abundance and protrusion into the extracellular space, the first bacterial structure that interacts with KP phages is very likely the CPS. In some bacterial species, phage infection is hindered by CPS presence [78], which acts as a passive barrier that hides other cellular receptors. In contrast, most KP phages are dependent on CPS presence to adsorb efficiently onto host cells [67]. *In vitro* evolution experiments in which KP strains were exposed to infectious phages revealed that resistance occurred most often through mutations that resulted in a lack of CPS production [79,80]. Even when a secondary phage receptor was required and exposed on the cell surface, phage infection was still hindered in the absence of CPS [70], suggesting that it is crucial for successful infection by many KP-targeting phages (Fig 1B).

**Fig 3:**
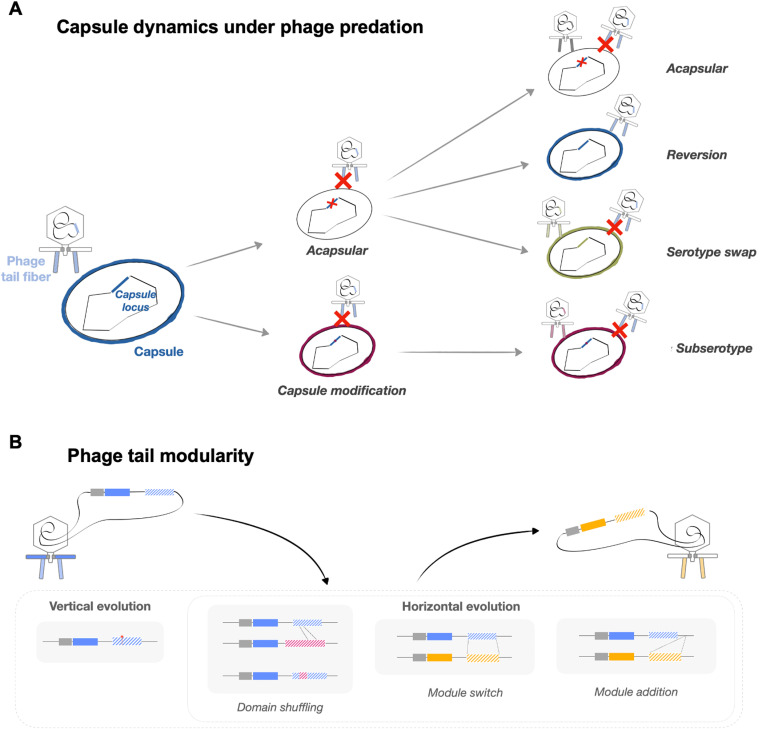
KP-targeting phage receptor dynamics. **(A)** Because most phages are dependent on CPS presence to adsorb efficiently, pressure from phage predation selects bacteria with reduced, altered, or no CPS production. Bacteria evolve to resist phages but become sensitized to other phages. **(B)** The arrangement of phage tail fiber and tailspike genes in a cassette-like organization enables rapid adaption to changes in bacterial epitopes. Phage specificity can rapidly evolve by mutating residues in the catalytic pocket (vertical evolution) or through horizontal gene transfer (HGT). HGT events between phages can result in the acquisition of enzymatic domains and the exchange of tailspike modules.

Because host tropism of KP phages appears to be mainly driven by CPS serotype [67,79] the infectivity of a given phage is likely limited to relatively few strains. The high specificity of phages for CPS has been used historically for serotype determination, as a complement to the traditional methods described above [81]. A recent study used 42 KP-targeting phages from various genera and tested their ability to infect 138 strains belonging to 59 different K-types [67]. The results showed that if a phage could infect one strain, there was a 92% chance it could also infect other strains with the same K-type. In agreement with this study, changing the K-type of a KP strain conferred resistance to phages that previously could infect the strain, and conferred susceptibility to phages to which the strain was formerly resistant [10] (Fig 3A). Other than CPS serotype, more subtle CPS variations also alter phage host range (Fig 3A). For example, insertion sequence (IS) disruption of the CPS locus or mutations in a putative acetyltransferase-encoding gene leading to reduced CPS acetylation both caused a change in phage host range and/or reduced phage adsorption [82,83]. Thus, even if CPS is properly expressed, fine-tuning of monosaccharide linkage or chemical modification can alter phage affinity for the capsular receptor.

While CPS-targeting KP phages are prevalent, the CPS is not the only phage receptor described [67,84]. Transposon-directed insertion site sequencing (TraDIS, a powerful tool to generate large loss-of-function mutant libraries) was used to identify alternative/secondary phage receptors essential for successful infection of KP and found that some phages required full-length LPS biosynthesis for infection [85] (Fig 1B). Similarly, mutations in the O-antigen biosynthesis genes *wecA* and *wecG* decreased phage adsorption and infection efficiency [86]. It remains unknown, however, whether LPS serves as a secondary phage receptor or if LPS is instead required for proper CPS assembly, anchoring, and/or positioning [13,85]. Because of the lower diversity of O-antigen types (Fig 2B), phages targeting LPS might be predicted to target a broader range of KP strains.

Beyond CPS and LPS, several surface-associated proteins have been identified as receptors for phage infection. These include the siderophore receptor FepA and the major porin OmpK36, commonly referred to as OmpC [70,87] (Fig 1B). Phage receptors can also be encoded on mobile genetic elements like plasmids, whereby some phages recognize specific components of the mating pair formation system to initiate adsorption [88,89] (Fig 1B). These plasmid-targeting phages can infect bacteria carrying IncF and IncP conjugative plasmids [88,89], many of which carry antibiotic-resistance genes [90,91]. A consequence of plasmid-dependent phage predation is that rather than targeting a particular bacterial strain, the phage exerts strong selective pressure against plasmid carriage and reduces dissemination throughout the population [92,93]. Given that antibiotic resistance and, more recently virulence factors [94], are known to be encoded on conjugative plasmids, such counterselection constitutes a beneficial by-product of phage therapy by reducing virulence [95,96].

## Host recognition by receptor-binding proteins and other phage tail modules

The major determinants of phage host range are RBPs, which are commonly located at the distal part of the phage tail (Fig 1C). A typical RBP of a KP-targeting phage is composed of three main sections: (i) an N-terminal domain that anchors it to the phage baseplate or another structural element of the tail, (ii) a C-terminal domain that acts either as an autochaperone or as a noncatalytic carbohydrate-binding module [85], and (iii) a mid-section β-helical domain with enzymatic activity, such as a depolymerase domain that cleaves surface polysaccharides (Fig 1C). Identification and characterization of phage RBP depolymerases is relatively recent [97,98], and knowledge about their diversity and mechanisms of action is scarce. For instance, it was previously believed that the trimeric state of tailspikes was crucial for enzyme stability, however recent biochemical studies showed that monomeric versions of the catalytic domain were also stable and active [99].

Phage RBP-encoded depolymerases cleave glycosidic bonds of polysaccharides, including CPS, LPS, or biofilm matrix, and thus facilitate the early steps of phage infection (Fig 1B). Phage depolymerases fall into two major categories: glycoside hydrolases, including O-antigen endoglycosidases and CPS endosialidases; and lyases, including pectate and alginate lyases that specifically cleave LPS, extracellular polymeric substances, CPS, or biofilm matrix [99]. Substrate specificity is determined by the depolymerase enzymatic pocket, which recognizes precise polysaccharide residues. Thus, even subtle changes in receptor structure or composition can confer phage resistance [83]. Despite this specificity, predicting depolymerase activity from RBP gene sequences alone is challenging because single mutations in the catalytic site can strongly impact enzymatic activity [99]. Additionally, different RBPs that can degrade the same polysaccharide can exhibit low sequence similarity, suggesting the use of alternative cleavage sites or convergent evolution [100].

Within phage genomes, tail fiber, tailspike, and lyase genes tend to be clustered and arranged in a cassette-like organization (Fig 3B). This organization likely facilitates rapid RBP evolution to modify residues in the catalytic pocket via horizontal gene transfer and recombination [101], resulting in the acquisition of new enzymatic domains or the exchange of tail modules between phages. The modularity of RBPs is predicted to enhance phage adaptability through rapid modification to expand the functional repertoire (Fig 3B), i.e. host range, thereby increasing phage fitness. A recent model proposed that anchor and enzymatic domains of RBPs could function as interchangeable building blocks [102], facilitating extensive mosaicism (Fig 3B) [103]. Such domain shuffling in RBPs appears to occur despite taxonomic and ecological barriers. The potential for varied combinations of RBPs appears to only be limited by the constraints posed by the virion assembly process. Most phages carry one or two RBPs with different depolymerase domains, thereby restricting their host range to only one or a few KP serotypes [104]. However, some phages carry multiple depolymerases targeting different K-types [84], such as the broad host range phage ΦK64-1 which encodes up to eleven depolymerases [100]. The fitness advantage of such generalist phages is strongly influenced by the ecological conditions and the severity of trade-offs, which can fluctuate over extended periods of co-evolution [105,106]. Indeed, a study in *Escherichia coli* showed that generalist phages with broad host ranges exhibited higher fitness compared to specialist phages with narrower host ranges, despite their slower adaptation rate [105]. This remains to be addressed in KP. Taken together, RBPs play a critical role in phage infection, and understanding how they interact with bacterial surface receptors can yield important new insights for phage therapy.

## Bacteria-phage dynamics: Evolving to escape from one another

**Fig 4:**
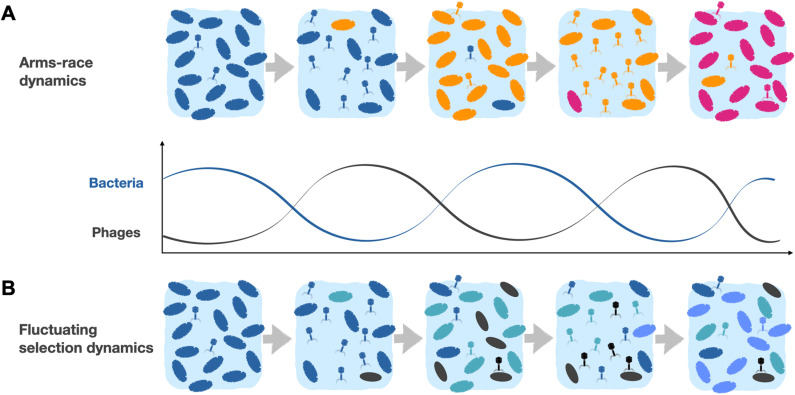
Models of co-evolution between bacterial and phage populations. **(A)** In the arms-race model, continuous adaptation by bacteria and phage leads to frequent selective sweeps and accumulation of new bacterial resistances and phage infectivities. **(B)** In the fluctuating selection model, phages maintain a narrow host range with infrequent selective sweeps. This enables the co-existence of multiple phage and bacterial genotypes, whose frequencies are driven by negative frequency-dependent selection and where rare genotypes have a fitness advantage.

Phages and bacteria are engaged in a co-evolutionary battle, with bacteria trying to resist phage infection and phages trying to infect their hosts more efficiently. While bacterial surface receptors and other defenses (i.e., anti-phage defense systems) have evolved to limit phage attacks, phages also diversify their targets through module shuffling and acquisition of new mechanisms to overcome bacterial defenses. There are two main models to explain phage-bacteria coevolution: “arms-race” dynamics and fluctuating selection dynamics [107] (Fig 4A, 4B). In the arms-race model, the continuous adaptation of both phage and bacteria leads to the accumulation of bacterial resistances and new phage infectivities (Fig 4A). Under this model, genotypes are replaced by successive selective sweeps that lead to phages with increased host ranges and bacteria with a large repertoire of phage resistance mechanisms. Evolved bacteria remain resistant to phages with ancestral traits, and evolved phages can still infect ancestral bacteria. Because of this, arms-race dynamics would very likely result in high fitness costs, ultimately leading to population extinction of either phage or bacteria.

On the other hand, the fluctuating selection model posits that phages evolve to overcome bacterial defenses at the cost of no longer being able to infect ancestral bacteria (Fig 4B). In this model, bacteria evolve to resist new phages, but in doing so, they may become newly sensitized to phages that they were previously resistant to. This is exemplified by K-type swaps, in which K-type exchanges allow a bacterial strain to resist infection by a given phage, but also result in sensitivity to other phages to which the strain was previously resistant [10]. The fluctuating selection model implies that phages maintain a narrow host range, with large selective sweeps being rare. Consequently, this model predicts a coexistence of numerous phage and bacterial genotypes whose dynamics are driven by negative frequency-dependent selection, wherein fitness changes over time as a function of allele frequency and rare genotypes have an advantage [108].

## Phage therapy for KP infections: Promise and challenges

Despite its use in Eastern Europe for nearly a century, phage therapy has emerged in Western medicine in the last decade as a potentially viable treatment approach for recurrent, recalcitrant, and multidrug-resistant bacterial infections. There are over a dozen reports of recent phage therapy treatment for KP infections in humans (Table 1). Successful reports include treatment of recurrent UTIs with KP-targeting phage cocktails [109,110] and clearance of KP biofilm in a prosthetic joint infection [111]. Additionally, over 30 studies have tested the treatment efficacy of phages using animal models of infection (Table 2), with most studies showing promising results. While phage therapy is often considered a last-resort salvage therapy for patients with no other viable treatment options, these therapeutic successes underscore the high potential of phages as next-generation antimicrobials.

**Table 1:** KP bacteriophage therapy studies in humans.

Study	Infection type	KP ST^1^	KP K-type^1^	Co-administered antibiotics	Phage(s) used (Family: Name)^2^	Phage dose (PFU/dose)	Administration route(s)	Outcome(s)
Kuipers and colleagues (2019) [153]	UTI	ND	ND	Meropenem	NR	NR	Oral; bladder irrigation; intravesical	Successful KP eradication
Bao and colleagues (2020) [109]	UTI	ST11	ND	Trimethoprim-sulfamethoxazole	NR: SZ-1, SZ-2, SZ-3, SZ-6, SZ-8, Kp165, Kp166, Kp167, Kp158, Kp169, Kp152, Kp154, Kp155, Kp164, Kp6377, HD001	2.5 × 10^10^	Bladder irrigation	Successful KP eradication
Corbellino and colleagues (2020) [113]	Permanent ureteral stent	ST307	ND	None	Tevenvirinae: vB_KpnM_GF	1 × 10^7^ (oral); 1 × 10^6^ (intrarectal)	Oral; intrarectal	Successful KP eradication
Rubalskii and colleagues (2020) [154]	Lung infection	ND	ND	Ceftazidime, Linezolid, Avibactam, Colistin, Meropenem, Cotrimoxazole, Tobramycin	Autographivirinae: KPV811 Tevenvirinae: KPV15	2 × 10^8^ (inhaled); 1.8 × 10^9^ (nasogastric)	Inhaled; nasogastric	Temporary KP eradication
Cano and colleagues (2021) [111]	PJI	ND	ND	Minocycline	NR: KpJH46Φ2	6.3 × 10^10^	IV	Resolution of symptoms
Qin and colleagues (2021) [155]	UTI	ST15	KL131	Piperacillin-Tazobactam	Podoviridae: JD902, JD907, JD908, JD910 Myoviridae: JD905	2.5 × 10^10^ (bladder); 5 × 10^9^ (kidney)	Bladder/kidney irrigation	Successful KP eradication
Rostkowska and colleagues (2021) [156]	UTI	ND	ND	Meropenem	NR	NR	Intrarectal	UTI cleared after nephrectomy
Zaldastanishvili and colleagues (2021) [157]	UTI	ND	ND	Metronidazole, Polymixin B, Neomycin	NR	NR	Oral; intravaginal	Temporary KP eradication
Doub and colleagues (2022) [158]	PJI	ND	ND	Ertapenem	NR: KP1, KP2	1 × 10^9^; 2 × 10^8^	IV	Resolution of symptoms
Eskenazi and colleagues (2022) [115]	Fracture infection	ST893	K20	Meropenem, Colistin	Tevenvirinae: vB_KpnM_M1	1 × 10^7^	Local instillation via catheter	Improved wound condition
Federici and colleagues (2022) [159]	IBD	ND	ND	None	Demerecviridae: 1.2–3 s Autographiviridae: MCoc5c	2.8 × 10^10^	Oral	Phage administered as Phase 1 clinical trial, no off-target dysbiosis observed
Le and colleagues (2023) [110]	UTI	ST307; ST3647; ST1015	KL102, K30, K21, K17	Ciprofloxacin	Tevenvirinae: Metamorpho, Mineola, pKp20	5 × 10^9^	IV	Successful eradication of bacterial burden, partial serum neutralization
Li and colleagues (2023) [112]	Lung infection	ST15	KL112	Amikacin, Ceftazidime-Avibactam	Podoviridae: Kp_GWPB35, Kp_GWPA139	>5 × 10^9^	Inhaled	Decreased bacterial load, resolution of symptoms

^1^Sequence types and K-locus types were provided in the published study, or genome sequences of reported KP strain(s) were analyzed with Kleborate.

^2^Phage subfamily information is reported instead of family name if available. Phage classification was reported as described in the published study and may not follow current genome-based classification schemes.

UTI, urinary tract infection; PJI, prosthetic joint infection; IBD, inflammatory bowel disease; ND, not determined; NR, not reported; IV, intravenous.

**Table 2:** KP bacteriophage therapy studies in mouse infection models.

Study	Infection type	KP ST^1^	KP K-type^1^	Phage(s) used (Family: Name)^2^	Phage dose (PFU/dose)	Administration route(s)	Administration timing
Hung and colleagues (2011) [160]	Liver abscess; Bacteremia	ND	K2	Podoviridae: NK5	2 × 10^5^–2 × 10^8^	Intragastric; Intraperitoneal	30 mpi; 6 hpi; 24 hpi
Kumari and colleagues (2011) [161]	Burn wound	ND	ND	NR: Kpn5	5 × 10^9^	Topical	Simultaneous
Gu and colleagues (2012) [162]	Bacteremia	ND	K2	Myoviridae: GH-K2 NR: GH-K1, GH-K3	3 × 10^4^–3 × 10^7^	Intraperitoneal	30 mpi
Cao and colleagues (2015) [163]	Pneumonia	ND	ND	Siphoviridae:1513	2 × 10^7^–2 × 10^9^	Intranasal	2 hpi
Chadha and colleagues (2016) [164]	Burn Wound	ST66	K2	Myoviridae: Kpn1, Kpn2, Kpn3, Kpn4, Kpn5	5 × 10^6^	Topical	7 dpi
Chadha and colleagues (2017) [165]	Burn Wound	ND	ND	NR: KØ1, KØ2, KØ3, KØ4, KØ5	NR	Liposome loaded; Intraperitoneal	30 mpi; 24 hpi
Anand and colleagues (2020) [166]	Pneumonia	ST375	K2	NR: VTCCBPA43	2 × 10^9^	Intranasal	2 hpi
Horváth and colleagues (2020) [167]	Peritonitis	ST15	K24	Siphoviridae: vB_KpnS_Kp13	1.75 × 10^8^	Intraperitoneal	10 mpi; 1 hpi; 3 hpi
Soleimani and colleagues (2020) [168]	Pneumonia	ND	ND	Myoviridae: vB_KpnM-Teh.1	1 × 10^8^–1 × 10^9^	Intraperitoneal	Simultaneous; 24 hpi
Dhungana and colleagues (2021) [169]	Peritonitis	ND	ND	Podoviridae: Kp_Pokalde_002	2.4 × 10^7^	Intraperitoneal; Oral	Simultaneous
Fayez and colleagues (2021) [170]	Burn Wound	ND	ND	Siphoviridae: ZCKP8	NR	Suspension or gel	7 dpi
Hesse and colleagues (2021) [171]	Bacteremia	ST258	KL107	Podoviridae: Pharr Siphoviridae: KpNIH-2	2.5 × 10^7^ or 5 × 10^7^	Intraperitoneal	1 hpi; 8 hpi; 24 hpi
Hao and colleagues (2021) [172]	Peritonitis	ND	K47	Autographivirinae: SRD2021	1 × 10^8^	Intragastric	12 hpi
Luo and colleagues (2021) [173]	Pneumonia	ND	ND	Siphoviridae: vB_Kpn_B01	2 × 10^8^	Intraperitoneal	4 hpi
Shi and colleagues (2021) [174]	Bacteremia	ST11	ND	NR: kpssk3	1 × 10^6^–1 × 10^7^	Intraperitoneal	3 hpi
Wang and colleagues (2021) [175]	Pneumonia	ND	K47	Myoviridae: vB_KpnM_P-KP2	1 × 10^7^–1 × 10^9^	Intranasal	1 hpi
Zhang and colleagues (2021) [176]	Pneumonia	ND	K20	Autographiviridae: vB_KpnP_Bp5	2 × 10^8^	Intraperitoneal	Simultaneous; 2 hours before infection; 2 hpi
Asghar and colleagues (2022) [177]	Bacteremia	ND	ND	Myoviridae: A¥L Siphoviridae: A¥M	NR	Intraperitoneal	Simultaneous
Bai and colleagues (2022) [178]	NR	ST2237	KL19	Myoviridae: vB_kpnM_17-11	5 × 10^8^	Injection	Simultaneous
Federici and colleagues (2022) [159]	GI tract colonization	ST323	KL21	Autographiviridae: Mcoc5c Demerecviridae: 1.2-3s, 8M-7 Myoviridae: PKP-55, KP-2-5-1	1 × 10^5^ and 1 × 10^9^	Oral	6 hpi; 9 hpi; 12 hpi
Gan and colleagues (2022) [179]	Pneumonia	ST11; ST383	ND	Podoviridae: pKp11 Siphoviridae: pKp383	1 × 10^9^	NR	2 hpi
Pu and colleagues (2022) [180]	Pneumonia	ST23	K1	Siphoviridae: BUCT541	2 × 10^4^–2 × 10^7^	Nasal drip	6 hpi
Singh and colleagues (2022) [181]	Bacteremia	ND	ND	Siphoviridae: KpBHU4, KpBHU14 Tectiviridae: KpBHU7	1 × 10^2^–1 × 10^5^, 1 × 10^10^	Intraperitoneal	Simultaneous; 6 hours before infection; 6 hpi
Volozhantsev and colleagues (2022) [182]	Soft tissue	ST86; ST493	K2	NR: KpV74	1 × 10^8^	Intraperitoneal	1 hour before infection; 3 hpi; 24 hpi
Fang and colleagues (2023) [183]	Pneumonia	ST259	K54	Autographiviridae: vB_KpnA_SCNJ1-Z Siphoviridae: vB_KpnS_SCNJ1-C Myoviridae: vB_KpnM_SCNJ1-Y	1 × 10^8^	Nasal drip	2 hpi
Gan and colleagues (2023) [184]	Steatohepatitis	ST1536	ND	Podoviridae: phiW14	1 × 10^4^–1 × 10^6^	Intragastric	Simultaneous
Ichikawa and colleagues (2023) [116]	Primary sclerosing cholangitis	ST37; ST145	K80; K3	Drexlerviridae: KP13-2 Stephanstirmvirinae: KP13-16 Straboviridae: KP13MC5-1 Autographiviridae: KP13MC5-2	Oral: 1 × 10^9^; IV: 1 × 10^8^	Oral; IV	Simultaneous
Liang and colleagues (2023) [185]	Bacteremia	ND	K54	Straboviridae: BL02	1 × 10^8^	Intraperitoneal	1 hpi
Rahimi and colleagues (2023) [186]	Pneumonia	ST273	K2	Drexlerviridae: PSKP16	2 × 10^7^	Nasal drip	30 mpi; 24 hpi
Tang and colleagues (2023) [125]	NR	ST86; ST23; ST489	K20; K1; K6	Slopekvirinae: FK1979	NR	NR	Simultaneous
Feng and colleagues (2024) [187]	Wound	ST11	KL64	Ackermannviridae: PH1/P01 Autographiviridae: P24 Drexlerviridae: P39	6 × 10^7^	NR	30 mpi
Kelishomi and colleagues (2024) [188]	Burn Wound	ST782	K2	Drexlerviridae: PSKP16	1.5 × 10^8^	Topical	2 hpi
Li and colleagues (2024) [189]	NR	ST307; ND	K5	Drexlerviridae: P1011	1 × 10^9^	Intraperitoneal	2 hpi
Tang and colleagues (2024) [190]	Bacteremia	ST86	K2	Slopekvirinae: FK1979 NR: phiR3	FK1979: 2 × 10^2^, 2 × 10^5^–2 × 10^8^, 2 × 10^11^ phiR3: NR	Intraperitoneal	2 hpi

^1^Sequence types and K-locus types were provided in the published study, or genome sequences of reported KP strain(s) were analyzed with Kleborate.

^2^Phage subfamily information is reported instead of family name if available. Phage classification was reported as described in the published study and may not follow current genome-based classification schemes.

GI, gastrointestinal; NR, not reported; ND, not determined; hpi, hours post-infection; mpi, minutes post-infection; dpi, days post-infection; IV, intravenous.

At the same time, the development of phage therapy for widespread use faces several challenges. First, there are several disconnects between phage studies conducted in animals and humans. While numerous animal studies have demonstrated the therapeutic efficacy of phages, these have largely focused on acute systemic infections like pneumonia and bacteremia, however, applications in humans have thus far targeted chronic infections like UTIs and joint infections (Tables 1 and 2). Extrapolating outcomes from animal studies to human patients can be complicated as chronic infections introduce additional challenges such as biofilm formation, development of phage-resistant mutants, or phage neutralization by the host immune system. These issues are not typically encountered in acute infections. Additionally, animal studies often use hypervirulent KP strains, while most patient case reports describe the treatment of classical and multidrug-resistant strains. Another challenge is the variety of phages used across different studies, as well as the use of single phages (i.e., monophage therapy) versus phage cocktails containing mixtures of distinct phages. Most clinical reports used phage cocktails to treat KP infections, and some suggest that this approach reduces the emergence of phage-resistant bacteria [109,110,112]. Other clinical reports have suggested that monophage therapy is sufficient to resolve KP infection [111,113]. The use of antibiotics in combination with phages in some studies also complicates the interpretation of study results and makes it challenging to determine the independent contribution of phages to infection clearance. Lastly, a lack of standardized treatment protocols and outcome measures makes it difficult to compare studies. Overall, while the available literature suggests that phage therapy has good efficacy and a favorable safety profile, well-controlled clinical trials are needed to robustly measure the broader utility of this therapeutic modality. No currently enrolling clinical trials are focused on KP infections specifically, however, the growing interest in this field may lead to the creation of such trials in the future.

An important step in developing phage therapy is determining the phages to be used. This determination is primarily based on phage host range. It is thought that the K-type specificity of most KP phages might be a double-edged sword; narrow host range enables the phages to target specific isolates while minimizing bacterial cross-resistance, however, the diversity of KP K-types likely reduces the overall species coverage of any individual CPS-targeting phage. To circumvent this limitation, there is increasing interest in CPS-independent phages. These phages recognize the O-antigen or surface-associated proteins, which tend to be more conserved across different KP strains, thus increasing phage host range [28,84,114]. *In vitro* evolution can also be leveraged to generate more efficient phages. One method involves preadapting phages by iteratively passaging them in the presence of a target KP strain to evolve a more active phage [115]. Another method uses *in vitro* evolution to generate KP strains that are resistant to an initial phage. These are then used as bait to isolate additional phages capable of targeting the phage-resistant KP strains. These additional phages can then be used in combination with the initial phage to create a cocktail that can target both the original KP strain and anticipated phage-resistant mutants [116]. Using phages that target different bacterial receptors and have different host ranges can also reduce the occurrence of phage resistance [117], which often evolves more rapidly *in vitro* compared with resistance to small molecule antibiotics [118,119]. Currently, alongside difficulties in the identification and production of suitable phages, obtaining regulatory approval for phage therapy can cause additional time delays between a compassionate use phage therapy request and the administration of phage to patients, a process that takes a median of 170 days [59]. These delays can be further lengthened in regions with limited resources, which lack access to phage therapy and often suffer from an increased burden of antimicrobial resistance.

The emergence of bacterial resistance to phage predation is often evoked as a concerning challenge to the potential success of phage therapy. While evolved phage resistance may limit therapeutic efficacy, it can also lead to beneficial trade-offs. For example, evolved phage resistance may result in increased bacterial antibiotic susceptibility, altered susceptibility to other phages, and changes in bacterial virulence [110,117,120]. Thus, even if phage therapy cannot directly clear KP infection, it can be used to steer the bacterial population toward a more treatable phenotype. Because KP-targeting phages typically rely on the CPS for adsorption, the emergence of phage resistance frequently involves the alteration or loss of the bacterial CPS (Fig 3A), which can have variable effects [87,102]. Acapsular KP variants have higher rates of conjugation and thus greater potential to acquire multidrug resistance [10]. CPS loss can also enhance tolerance to membrane-targeting antimicrobial peptides [121] allowing bacterial regrowth even under high-dose antibiotic treatment. Furthermore, a recent report found that *in vitro* phage exposure led to the formation of KP persister cells that had a 6-log increase in survival when exposed to lethal concentrations of antibiotics [122]. These persister cells also slow the pace of bacteria-phage coevolution and selection [123], promote anti-phage defenses [124], and evade antibiotic killing, thereby enabling regrowth post-treatment. On the other hand, acapsular KP variants have decreased rates of gastrointestinal tract colonization and diminished virulence compared with encapsulated strains [112,125]. Overall, understanding trade-offs driven by phage exposure and evolution of phage resistance can inform the development of phage therapies, alone or in combination with antibiotics, that effectively leverage these trade-offs for maximal therapeutic benefit.

## Looking ahead: The future of phage therapy for KP infections

**Fig 5:**
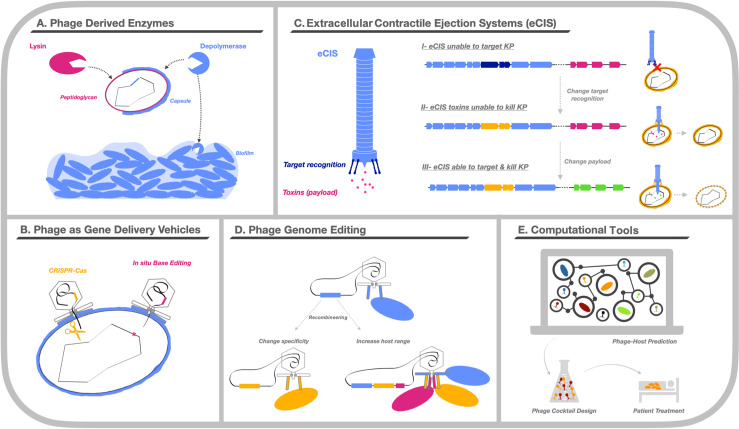
Looking ahead—The future of phage therapy for KP infections. **(A)** Phage-derived enzymes like lysins and depolymerases can degrade peptidoglycans and carbohydrates, including those in the bacterial CPS and KP biofilm matrix. **(B)** Phages could be used as gene transfer agents to deliver predetermined “cargo” to bacterial cells and use CRISPR-Cas systems to kill directly or edit the bacterial genome. **(C)** Extracellular Contractile Ejection Systems (eCIS) are syringe-like macro-molecular systems that deliver toxins into adjacent cells. eCIS could be reprogrammed to change their specificity and/or express alternative payload molecules to combat bacterial infections. **(D)** Phage genome editing can be performed through a process called “recombineering”. Recombineering enables modification, reduction, or broadening of phage host range. **(E)** Computational tools are being developed to predict interactions between phages and potential bacterial hosts. Machine learning and modeling allow rapid identification of candidate phages for a given bacterial infection, enabling the design of highly specific and optimized phage cocktails for use in clinical settings.

With the growing popularity of phage therapy, there is also increased interest in phage-derived strategies, such as phage enzymes with antibacterial properties. These enzymes, namely lysins and depolymerases, have shown promise in combatting bacterial infections, with little to no adverse effects (Fig 5A) [126,127]. Lysins are phage-encoded enzymes that digest the peptidoglycan of bacterial cell walls. Studies have shown that exogenous addition of lysins exhibits antibacterial activity both *in vitro* and *in vivo* [128]. Additionally, resistance to lysins is rare, likely due to their targeting of a highly conserved region of the cell wall [129,130]. Several lysins with activity against KP have been described [131,132]. Depolymerases, on the other hand, degrade carbohydrates and exhibit high substrate specificity for CPS, LPS, or other extracellular polysaccharides. These enzymes have therapeutic potential as standalone agents, as they can degrade KP biofilms and make the bacteria more sensitive to antimicrobials or the immune system, thus promoting infection clearance [133–135]. However, the large molecular mass of depolymerases may limit their tissue penetration, and as proteins, they are likely to stimulate an immune response and might prompt the generation of neutralizing antibodies that would likely reduce their effectiveness over time. Additionally, the effectiveness of depolymerases, as with phage therapy in general, can be limited by the emergence of resistance due to modifications or variations in bacterial surface-associated polysaccharides.

Phages can also be used as gene transfer agents that can deliver pre-determined “cargo” to bacterial cells (Fig 5B). Initial efforts have focused on the delivery of CRISPR-Cas systems that can either kill bacteria outright or eliminate undesirable genes from the bacterial population [136]. More recently, a phage-derived particle was used to perform *in situ* base editing of *E. coli* and KP colonizing the mouse gut [137]. Additionally, extracellular contractile injection systems (eCISs) have been described as an additional phage-derived antimicrobial system (Fig 5C). These are syringe-like macro-molecular systems that deliver toxins into adjacent cells and appear to have evolved from bacteriophage tails [138–140]. Like phages, these systems recognize specific receptors in the target cell and subsequently release a broad range of toxins that inhibit microbial growth. Recent studies have shown that eCISs can be “reprogrammed” and engineered to deliver a variety of different payloads in a strain-specific manner. Given the large number of eCISs currently described (>1,200) [139], these systems could constitute a novel and untapped source of phage-based antimicrobial strategies for further development. The potential of these phage-derived strategies to degrade KP biofilms and sensitize bacteria to other antimicrobials are particularly attractive features, thus their characterization and further development warrants additional study.

As modern medicine becomes increasingly personalized, the development of phage therapy has prompted the use of phage genome editing (Fig 5D). These techniques have largely focused on engineering genomes through recombination (i.e., recombineering), initially through the use of phage lambda as a model system to integrate linear DNA into the viral genome [141,142]. Additional methods such as BRED (bacteriophage recombineering of electroporated DNA) have been developed to facilitate genome manipulation and precise mutation of phage genes [143,144]. The integration of the CRISPR-Cas system has proven effective in both enhancing recombination efficiency [145] and selectively editing phage genomes [146]. Furthermore, plasmids encoding lambda-red recombinase have been employed as a strategy to further increase recombination efficiency [100]. These diverse recombineering approaches represent a substantial leap forward in the field of phage genome modification and pave the way toward finer specificity of phage therapy to modulate phage host range.

An alternative approach to phage genome editing is the use of computational approaches to rapidly predict and identify suitable phages based on bacterial and phage genome sequences (Fig 5E). Two recently developed computational tools to predict interactions between phages and potential bacterial hosts, iPHoP [147] and CHERRY [148], aim to accurately predict an individual phage’s host at the genus and species level, respectively. In the context of phage therapy, however, prediction of activity at the strain level is likely required. While initial studies of *in silico* prediction of KP depolymerase specificity showed some uncertainty and generated many incorrect predictions [99], a more recent *in silico* RBP protein clustering-based method accurately forecasted a majority of productive infections in KP [67]. These predictions were limited to tropism driven by CPS type, however, and did not account for alternative KP receptors or phage resistance post-adsorption. Nonetheless, it appears that adsorption factors alone could be sufficient to predict many phage–bacteria interactions [149,150]. Expansion of these tools with larger collections of phages and KP strains would be beneficial to increase their accuracy and robustness.

The implementation of machine learning and modeling approaches in experimental labs, and potentially in the clinic, also opens new possibilities for the design of effective phage-based therapeutics. For example, recently developed algorithms designed to determine optimal phage cocktails to target specific *E. coli* strains based on predicted phage–bacteria interactions could be easily adapted to KP [150]. Additionally, a model-based approach using experimental data for four different multidrug-resistant KP isolates was recently used to select optimal combinatorial phage regimes [151]. When functionally tested, the predicted regimes were able to effectively reduce bacterial loads to a pre-specified target threshold. Access to automated computational pipelines could help design optimized strategies that take into account a large number of variables, including but not restricted to: phage RBPs, inter-phage interactions [152], the presence and expression of phage receptors on targeted pathogens, pharmacokinetics, pharmacodynamics, and patient-specific factors. We expect that the development of automated methods to predict highly specific and optimized phage cocktails will pave the way toward large-scale, precise, and personalized phage therapy.

## Conclusions

The dramatic increase in multidrug-resistant KP strains worldwide, as well as their increasing convergence with hypervirulent traits, calls for new strategies to fight these worrisome infections. The phage therapy field is booming, and the resulting enthusiasm should be harnessed to propel the field forward to develop therapeutically effective protocols for clinical applications. Open-source initiatives, community engagement, and active crosstalk between researchers and clinicians are also crucial to bring phage therapy out of the laboratory and into the clinic. Standardized procedures and testing, rational therapeutic design, and leveraging the power of predictive computational tools will all facilitate this process. Additionally, the integration of evolutionary approaches and mathematical modeling with clinically relevant observations can help increase our understanding of what will make phage therapy an effective antimicrobial strategy. We are working toward a future where we can reliably predict the evolutionary trajectories of individual bacterial hosts upon exposure to phage predators, and can harness trade-offs of phage resistance to limit bacterial virulence and potentiate the effects of antibiotics and the immune system. We hope that rationally designed phage therapies will soon be possible and that they will improve the treatment and control of KP infections around the world.
